# Machine Learning-Based Co-Expression Network Analysis Unravels Potential Fertility-Related Genes in Beef Cows

**DOI:** 10.3390/ani12192715

**Published:** 2022-10-09

**Authors:** Wellison J. S. Diniz, Priyanka Banerjee, Soren P. Rodning, Paul W. Dyce

**Affiliations:** Department of Animal Sciences, Auburn University, Auburn, AL 36849, USA

**Keywords:** biomarker, cow fertility, data mining, machine learning, transcriptomics

## Abstract

**Simple Summary:**

Female reproductive failure is still a challenge for the beef industry. Several biological processes that underlie fertility-related traits, such as the establishment of pregnancy and embryo survival, are still unclear. Increased availability of transcriptomic data has allowed a deep investigation of the potential mechanisms involved in fertility. This study investigated candidate gene biomarkers predictive of pregnancy status and underlying fertility-related networks. To this end, we integrated gene expression profiles through supervised machine learning and gene network modeling. We identified nine biologically relevant endometrial gene biomarkers that could discriminate against pregnancy status in cows. These biomarkers were co-expressed with genes critical for uterine receptivity, including endometrial tissue remodeling, focal adhesion, and embryo development. This study outlined key pathways involved with pregnancy success and provided predictive candidate biomarkers for pregnancy outcome in cows.

**Abstract:**

Reproductive failure is still a challenge for beef producers and a significant cause of economic loss. The increased availability of transcriptomic data has shed light on the mechanisms modulating pregnancy success. Furthermore, new analytical tools, such as machine learning (ML), provide opportunities for data mining and uncovering new biological events that explain or predict reproductive outcomes. Herein, we identified potential biomarkers underlying pregnancy status and fertility-related networks by integrating gene expression profiles through ML and gene network modeling. We used public transcriptomic data from uterine luminal epithelial cells of cows retrospectively classified as pregnant (P, n = 25) and non-pregnant (NP, n = 18). First, we used a feature selection function from BioDiscML and identified *SERPINE3*, *PDCD1*, *FNDC1*, *MRTFA*, *ARHGEF7, MEF2B*, *NAA16,* ENSBTAG00000019474, and ENSBTAG00000054585 as candidate biomarker predictors of pregnancy status. Then, based on co-expression networks, we identified seven genes significantly rewired (gaining or losing connections) between the P and NP networks. These biomarkers were co-expressed with genes critical for uterine receptivity, including endometrial tissue remodeling, focal adhesion, and embryo development. We provided insights into the regulatory networks of fertility-related processes and demonstrated the potential of combining different analytical tools to prioritize candidate genes.

## 1. Introduction

The sustainability of a cow-calf production system relies on the efficiency of reproductive performance per cow. However, a decline in cattle fertility has led to increased reproductive failure [1,2], which is a challenge for beef producers and a significant cause of economic loss [3]. Fertility is a lowly heritable, multifactorial trait affected by genetic, environmental, and management factors [2,4,5]. Despite the limited selection response to traditional selective breeding strategies, reproductive and genomic technologies have provided opportunities to improve reproductive efficiency [3,6]. Several candidate genes and biological processes have been identified through genome-wide association studies (GWAS) [7,8,9]. Likewise, genomic testing and selection, mainly in dairy cattle, has increased the rate of genetic improvement for female fertility [2,8].

Other omics approaches, such as transcriptomics and metabolomics, have shed light on the complex regulatory mechanisms of cattle fertility [10,11,12,13,14]. Canovas et al. [12] reported 1515 differentially expressed genes (DEGs) from eight tissues by comparing pre- and post-pubertal Brangus heifers. Similarly, Geary et al. [14] profiled the endometrium of highly fertile, subfertile, or infertile heifers and found downregulated DEGs involved with immune response. From metabolomic approaches, ornithine and L-alanine were less abundant in the blood plasma of infertile cows and were reported as potential biomarkers of pregnancy outcome through artificial insemination [11] or embryo transfer [15]. Despite the fruitful results and the massive amount of information provided by different omics approaches, no major gene or causal mutation determining fertility-related traits has been reported. Additionally, most studies have focused on differential expression analysis. Despite its benefits, this approach does not account for the complex interactions among genes. Thus, a systemic approach considering the multiple gene relationships can add new knowledge to decipher the gene architecture of female cattle fertility. 

Several analytical methods have been proposed to mine meaningful biological information from complex biological data. Genes do not work alone. Thus, gene network approaches have been used to reduce the dimensionality of omics data and outline specific molecular mechanisms driven by co-expressed genes [16,17]. Likewise, ML methods have provided opportunities to dissect genomic signatures from omics data [18]. ML is a broad term encompassing different methods that use self-learning algorithms to analyze large, complex data and extract patterns that can be used for prediction [18,19]. Despite the opportunities provided by these methods, few studies have coupled these tools to address the interplay between genes and fertility. Based on ML, Rabaglino et al. [19] integrated endometrial transcriptomic profiles from public datasets and identified 50 genes as predictors of uterine receptivity to embryo transfer in cattle. Fonseca et al. [20] reported 32 functional candidate genes from a co-expression network meta-analysis by comparing transcriptomic profiles of high and low-fertile crossbred heifers. Combining blood transcriptomics, ML, and gene networks, Moorey et al. [10] reported *RPL39, SMIM26, LONRF3, GATA3,* and *N6AMT1* as the top five genes for classification of pregnancy outcome at the artificial insemination timepoint in heifers. 

In this work, we presented a comprehensive multi-tiered approach using ML feature selection, gene co-expression network, and functional analysis on transcriptomic profiles of uterine luminal epithelial cells. Specifically, ML was assigned to screen out gene expression signatures to predict whether a recipient cow would become pregnant or remain open. Furthermore, a gene co-expression framework was used for modeling gene relationships and putative regulatory mechanisms involved with fertility and pregnancy outcomes. Our goal was to identify potential gene biomarkers predictive of pregnancy outcomes and underlying fertility-related networks by integrating gene expression profiles and prioritizing candidate genes retrieved through ML and gene network modeling. We have demonstrated the potential of combining different methods to identify candidate biomarkers and provided insights into the complex genomic basis underlying pregnancy establishment and fertility in cattle. We identified nine biomarkers discriminating between P and NP cows, including *SERPINE3*, *MRTFA*, and *ENSBTAG00000019474.*

## 2. Materials and Methods

All transcriptomic and phenotypic data used in this current study were retrieved from the Gene Expression Omnibus database (GEO – Accession number GSE171577, BioProject PRJNA720121). The dataset comprises RNA-sequencing profile, progesterone level, block, and pregnancy status of 43 multiparous, Angus-Brahman crossbred cows. 

**Figure 1:**
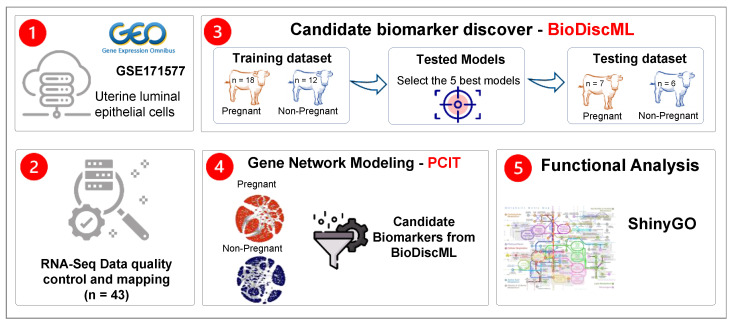
Overview and analysis workflow to identify predictive biomarkers of pregnancy status and fertility-related networks in cows.

The data and the main findings were published by Martins et al. [21]. In brief, estrous synchronized recipient cows had uterine luminal epithelial cells sampled three days before embryo transfer. Pregnancy was diagnosed on day 30 through transrectal ultrasonography. Pregnancy status was as follows: 25 pregnant (P) and 18 non-pregnant (NP) cows. Further information about experimental design and laboratory procedures is described elsewhere [21]. An overview of the methodological approach for the current study is shown in Figure 1. 

### 2.1. Data Retrieval and Quality Control 

The FASTQ files were downloaded from the GEO database using a bash script from the SRA-Explorer web tool [22]. Data quality control was carried out using FastQC v0.11.9 (https://bit.ly/3pCUvar, accessed on 6 January 2022) [23] and MultiQC v1.11 (https://multiqc.info/, accessed on 6 January 2022) software [24]. On average, each sample had 23.7 M reads (paired-end, 150 bp) with a PhredScore greater than 35. The *Bos taurus* genome ARS-UCD 1.2 [25] was used as the reference for sequence assembly and annotation. Read mapping was performed using the two-pass mode of the STAR aligner v.2.7.5 (https://rb.gy/dlgdva, accessed on 6 January 2022) [26]. The –*quantMode GeneCounts* flag from STAR and the annotation file (release 104) from *Ensembl* were used for read counting. Post-mapping quality control was performed using MultiQC, Principal Component Analysis (PCA) using the R software [27], NOISeq v.2.38.0 (10.18129/B9.bioc.NOISeq, accessed on 6 January 2022) [28], and edgeR v.3.36.0 (10.18129/B9.bioc.edgeR, accessed on 6 January 2022) [29] R-packages. 

### 2.2. Gene Expression Normalization and Supervised Machine Learning

Counts for unstranded RNA-Seq for each sample were retrieved from STAR and transformed to counts per million (CPM) using edgeR. Genes with low count expression (CPM < 0.5 in 50% of samples) were filtered out [29]. The gene expression normalization procedure used the DESeq2 v.1.26.0 (10.18129/B9.bioc.DESeq2) *VST* function. The gene expression values were adjusted for the effect of the block, as reported by Martins et al. [21], using the *removeBatchEffect* function from the Limma R-package [30]. 

The VST normalized genes were subjected to ML using BioDiscML. BioDiscML automates ML steps by implementing methods for features (genes) and model selection [31]. To compare the prediction performance of the models, we ran the software on the training dataset using a categorical classification (pregnant or non-pregnant—P or NP). To this end, 2/3 of the samples (n = 30; 18 P and 12 NP) were randomly used for training. The remaining cows (n = 13; 7 P and 6 NP) were used for model validation. Then, the feature ranking algorithm sorted the features based on their predictive powers with respect to class (P or NP). Based on that, only features (genes) with an information gain > 0.01 were selected for further analysis [31]. Two methods were used by the software for model selection: top k features and stepwise for each algorithm and each optimization evaluation criteria. The models generated were evaluated by tenfold cross-validation and the genes improving the predictive performance were retained. Once the models were optimized, prediction performance was measured using 10 cross validations (CV), leave-one-out cross validation (LOOCV), holdout, repeated holdout, bootstrapping, and a 0.632+ bootstrap estimator [31].

Since the software generates many models, we selected the top five (*numberOfBestModels* = 5). In the current dataset, the classifier was categorical (P or NP). Therefore, a stable model selection and evaluation method that minimized the overfitting of the data was used. For this purpose, the software recommends selecting a model having the best average Mathew’s correlation coefficient (AVG_MCC) with a standard deviation lower than 0.1. The Mathew’s correlation coefficient provides an informative and truthful score in evaluating binary classifications [32]. The top five best models were selected using the *numberOfBestModelSortingMetric* as AVG_MCC on the training set. The test dataset was validated using the *predict* function with the genes from the top 5 selected models. These genes were identified as potential biomarkers and subjected to further analysis. The expression differences of the selected candidate biomarkers between the P and NP groups were visualized using the ggplot2 v3.3.5 R-package [33]. 

### 2.3. Gene Co-Expression Network Analysis 

To investigate the coordinated gene expression and putative regulatory relationships underlying the differences between P and NP cows, we created two independent networks. Thus, the normalized genes (see Methods Section 2.2) were used. To create the networks, we used the Partial Correlation and Information Theory (PCIT) algorithm [34], as described by Diniz et al. [35]. This approach explores relationships between all possible triplets of genes to determine truly informative correlations between gene pairs [17,34]. 

Significantly correlated pairs were selected when the candidate biomarkers from the ML approach were identified (*p* ≤ 0.05). The Network Analyzer tool [36] was used for network analysis, and Cytoscape v.3.8.2 [37] was used for visualization. The highly connected genes, or “hubs”, were identified considering the degree measure (Mean + 2SD) retrieved from Network Analyzer. The changes in the nodes and edge rewiring from the P and NP groups were visualized using DyNet [38]. To identify the differentially connected genes in each group, the connectivity (K) measure for each network was standardized by dividing the gene connectivity by the maximum connectivity [39]. The differential connectivity (DK) measure was calculated as $DK_{i}=K_{NP\left(i\right)}-K_{P\left(i\right)}$. The DK values were transformed to a z-score and values ± 1.96 SD were considered significant (*p* ≤ 0.05) [35]. The networks were visualized using Cytoscape v3.8.2. 

### 2.4. Functional Over-Representation Analysis 

Functional over-representation analysis was performed to retrieve biological processes based on gene ontology (GO) terms and KEGG pathways that underlie the co-expressed genes. The queried gene lists included the biomarker-gene co-expressed pairs within P and NP cows separately. Likewise, overlapping genes were analyzed to identify shared pathways. Functional annotation of unknown genes was based on the targeted co-expressed genes. The over-representation analyses were performed using ShinyGO v0.76 [40], which calculates a hypergeometric test followed by a false discovery rate (FDR) correction. A customized background gene list (Table S1) for functional analysis was based on all expressed genes detected in our dataset (n = 15,039). Significant results were retrieved after *p*-*value* adjustment using the Benjamini–Hochberg method (FDR ≤ 0.05).

## 3. Results

In this study, our goal was to investigate potential gene biomarkers underlying pregnancy status and fertility-related networks by integrating gene expression profiles through supervised machine learning (ML) and gene network modeling. First, we used a feature selection function from BioDiscML and identified genes as candidate predictors of pregnancy outcome. Then, we created gene co-expression networks to identify differences in network topology linked to the gene predictors identified through ML. Lastly, we used the co-expressed networks to get insights into the gene function and over-represented biological processes affecting fertility as measured by pregnancy status. 

Figure 1 summarizes the approach adopted in the current study. After QC, on average, 93.4% of unique reads were mapped to the bovine reference genome. A summary of sequencing throughput and read mapping per sample is available in Table S2. Based on the QC criteria, 15,039 genes out of 27,607 were kept for further analysis (see Section 2.2). 

### 3.1. Identification of Potential Biomarker Genes through ML 

The ML steps were automated by BioDiscML for feature and model selection on 15,039 genes. The feature ranking algorithm sorts the genes based on predictive powers based on class (P or NP) and retains the genes with an information gain > 0.01. Considering the predictive power with respect to class exhibited by the feature ranking algorithm, we retrieved 225 genes for model evaluation with an information gain > 0.01. Based on that, 4524 models were generated out of 6580 models tested by the software. Next, we selected the top 5 models with an AVG_MCC > 0.97 (see Methods Section 2.2) to evaluate the test dataset (Table S3). These models were from three categories: functions, lazy, and rules. The details of the models are as follows:

(1). Functions category with SPegasos (Stochastic primal estimated sub-gradient solver for SVM) as classifier optimized by false discovery rate (FDR);

(2). Three models of the lazy category with IBk (K-nearest neighbors with and without Gaussian) optimized by Matthew’s correlation coefficient (MCC), FDR, and balanced error rate (BER) as the classifier;

(3). Rules category with ordinal learning method (OLM) as the classifier and optimized by the area under the curve (AUC).

In the training dataset, all the models, except for rules-OLM-AUC, exhibited a prediction accuracy greater than 90%. The accuracy considered the following evaluation procedures: tenfold cross-validation, leave-one-out cross-validation, repeated holdout, and bootstrapping in the entire dataset. For the same evaluation procedures, the rules-OLM-AUC model exhibited 80% accuracy on the training dataset. 

**Figure 2:**
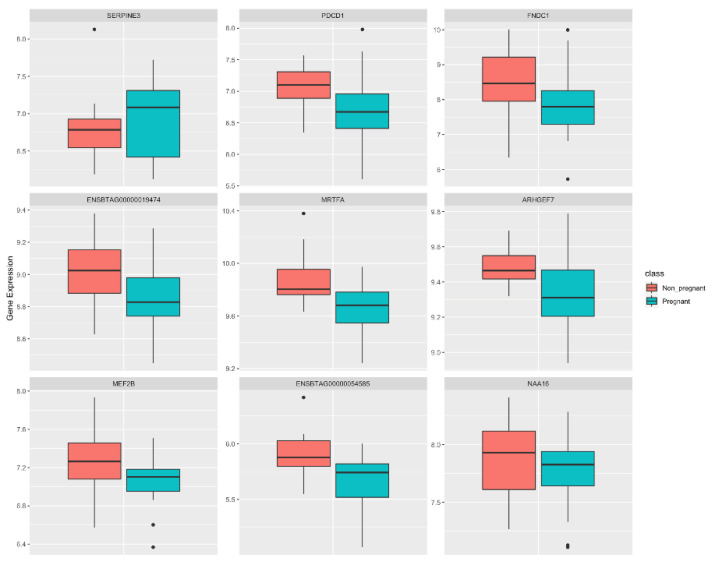
Normalized gene expression of candidate biomarkers discriminating between pregnant (P) and non-pregnant (NP) cows. Boxplot limits are associated with the first (lower) and third (upper) quartiles. Horizontal lines within the boxplots represent the median of normalized expression data for each cohort (P and NP). Black dots outside the vertical range of whiskers represent outliers.

Using these models, nine genes (*SERPINE3*, *PDCD1*, *FNDC1*, *MRTFA*, *ARHGEF7, MEF2B*, ENSBTAG00000019474, ENSBTAG00000054585, and *NAA16*) were identified as discriminating between P and NP cows and are reported here as candidate biomarkers. Figure 2 shows the differences in the expression levels of the nine candidates between the P and NP groups. To evaluate the prediction performance of the identified models based on the candidate biomarkers, we tested each model on the validation dataset and the accuracy was recorded. All the models exhibited an accuracy of 61.54%, except for the rules-OLM-AUC model (accuracy = 53.85%). 

### 3.2. Gene Network Analysis 

**Figure 3:**
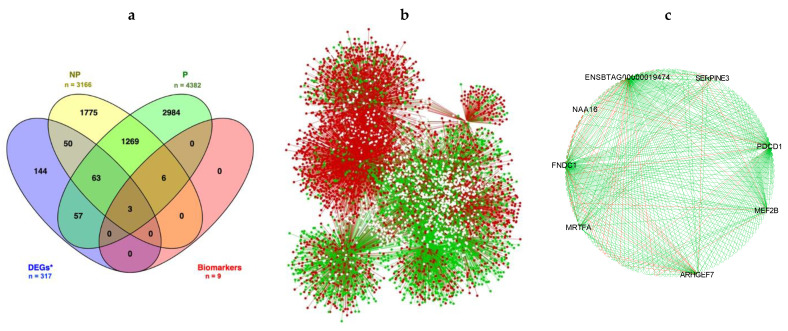
Uterine luminal epithelial co-expressed genes between pregnant (P) and non-pregnant (NP) cows. (**a**) Genes that overlap across analyses; *DEGs – differentially expressed genes from Martins et al. [21]; Biomarkers were identified through machine learning. (**b**) Central reference union networks between the P and NP groups, with 6202 nodes (genes) and 9020 edges (interactions); (**c**) Only gene pairs that are co-expressed with a candidate biomarker are shown; red and green lines (connections) represent negative and positive correlation, respectively.

To investigate the functional gene-gene relationship between P and NP cows, we created co-expression networks from 15,039 genes for each group separately using PCIT. Using this approach, we identified 8,554,787 and 7,227,015 significantly correlated pairs (*p* < 0.05) for P and NP, respectively. To reduce the data dimensionality, we kept only gene pairs correlated with the candidate biomarkers (nine genes as identified above). Thus, 5412 and 4204 pairs were kept (corresponding to 4382 and 3166 unique genes) in the P and NP networks, respectively (Figure 3, Table S4). By overlapping the gene lists, we identified 1341 genes that were shared between the groups (Figure 3a). To visualize the connectivity between P and NP gene networks, we built a central reference network using DyNet. This network comprised 6202 nodes (genes) and 9020 edges (interactions) (Figure 3b). The nodes were filtered for the nine candidate biomarkers based on degree measure (see Methods Section) from both the P and NP groups (Figure 3c).

Considering the differential connectivity measure, we identified seven genes significantly rewired (gaining or losing connections) between P and NP networks (Figure 3c, Table 1 and Table S5). Despite the similar topological behavior, the P network had more co-expressed nodes than NP. On the other hand, the candidate biomarkers identified in the NP cows were more connected. A significant increase in connectivity in the NP networks was identified for the *MEF2B, FNDC1, ENSBTAG00000019474, SERPINE3,* and *MRTFA* genes. Conversely, *NAA16* and *AEHGEF7* were more connected in the P network.

Further, we examined the DEG list from Martins et al. [21] to investigate whether these genes were co-expressed with the candidate biomarkers we found. By overlapping the lists, we identified 66 genes that were shared between the P, NP, and DEG lists (Figure 3a). Genes that were DEGs [21], identified as biomarkers through ML, and differentially connected included *ENSBTAG00000019474,* *PDCD1,* and *MRTFA*. 

### 3.3. Functional Over-Representation Analysis

**Figure 4:**
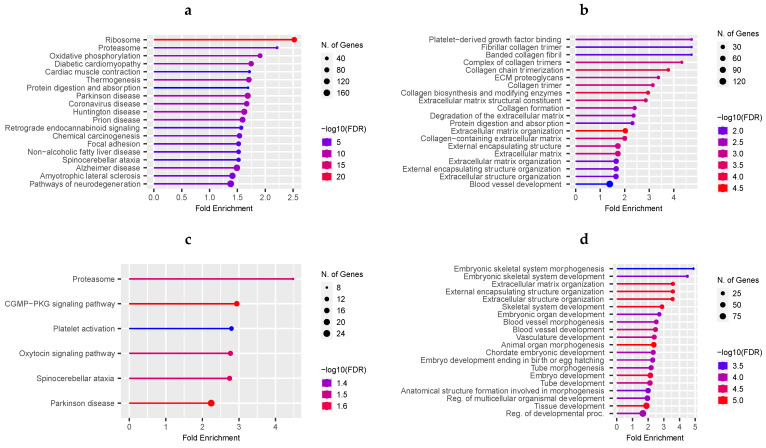
Top over-represented KEGG pathways and biological processes (BP) underlying uterine luminal epithelial co-expressed genes. KEGG pathways from the individual network of co-expressed genes from pregnant (**a**) and non-pregnant cows (**b**); BP of individual subnetworks of genes co-expressed with the ENSBTAG00000019474 gene from pregnant (**c**) and non-pregnant cows (**d**).

We used a functional over-representation analysis based on the ShinyGo tool to retrieve biological processes and KEGG pathways affected by the co-expressed genes. The over-representation analyses of overlapping genes between P and NP groups (n = 1341) retrieved protein digestion and absorption, ECM-receptor interaction, and focal adhesion (FDR < 0.05). Additionally, we analyzed the gene lists separately for P (n = 4382) and NP (n = 3166) (Figure 4a,b). Unique pathways from pregnant co-expression networks included ribosomes, proteasomes, and oxidative phosphorylation (Figure 4a, Table S6). Likewise, pathways related to tissue remodeling, such as degradation of the extracellular matrix, collagen formation, ECM proteoglycan, and blood vessel development, were over-represented by co-expressed genes from the NP network (Figure 4b, Table S7). 

To predict the biological processes (BP) of the ENSBTAG00000019474 and ENSBTAG00000054585 identified as candidate biomarkers, we used their respective co-expressed genes. The queried list of the ENSBTAG00000019474 included 508 and 578 unique genes, whereas the ENSBTAG00000054585 gathered 145 and 47 from P and NP subnetworks, respectively. The significant over-represented BP (FDR < 0.05) underlying the co-expressed genes for P and NP cows from the ENSBTAG00000019474 gene are shown in Figure 4c,d (Tables S8 and S9). In the NP subnetwork, most of the genes were positively correlated (413 pairs out of 577) with ENSBTAG00000019474. Interestingly, the most over-represented terms were those related to early pregnancy and included embryo development, tissue development, and vasculature development (Figure 4d). No significant BP was over-represented for the genes co-expressed with ENSBTAG00000054585.

## 4. Discussion

Fertility is a general term encompassing a variety of traits important in animal reproduction [41]. Herein, we will broadly define fertility as “the ability to conceive and maintain a pregnancy” [2,41]. The establishment of pregnancy and embryo survival are dependent on a cascade of biochemical and hormonal events. Thus, the uterine environment is critical in supporting pregnancy [42]. Likewise, changes in gene expression of endometrial epithelial cells are associated with pregnancy success or failure [21]. In this study, we investigated candidate gene biomarkers underlying fertility-related networks and pregnancy outcomes by integrating gene expression profiles through supervised machine learning and gene network modeling. To this end, we used public transcriptomic data generated from uterine luminal epithelial cells of recipient cows biopsied three days before embryo transfer [21]. The data was published by Martins et al. [21], which reported 240 genes affected by progesterone concentration and 317 differentially expressed genes (DEGs) by comparing P and NP cows based on a linear model. Furthermore, the authors identified 25 genes with a predictive ability to discriminate against pregnancy outcomes [21]. 

Pregnancy success is dependent on a number of events that include embryonic viability and a receptive uterine environment to sustain embryonic growth and development [43,44]. Regarding embryonic viability, Martins et al. [21] transferred either fresh or frozen embryos to recipient cows. Although the pregnancy rate per embryo transfer has previously been shown to be lower for recipients receiving frozen embryos [45], no significant differences in pregnancy rates were reported by Martins et al. [21]. Several studies have focused on endometrial biopsies to profile gene expression and characterize the mechanisms underlying endometrial receptivity [14,43,44,46]. In the current study, however, the authors proposed a less invasive approach and sampled luminal epithelial cells using a cytobrush [47]. While there are tissue-specific differences [47], the gene expression profile of epithelial cells provides a representative picture of the physiological status of the uterine environment [21]. 

Herein, we have shown the potential of combining different analytical tools to prioritize candidate genes. Our results, however, should be interpreted considering the limited sample size used to train and predict ML models. Additionally, the expression profile was measured at one timepoint in only one tissue and does not capture all the genomic mechanisms and factors involved with pregnancy success [10]. Lastly, we cannot expect the expression of a limited number of genes to provide high accuracy in determining pregnancy outcomes [19]. Thus, testing in larger cohorts with a similar approach would provide increased accuracy and reliability of the genes as predictors.

Based on the ML approach, we identified *SERPINE3*, *PDCD1*, *FNDC1*, *MRTFA*, *ARHGEF7, MEF2B*, ENSBTAG00000019474, ENSBTAG00000054585, and *NAA16* as candidate biomarkers discriminating between P and NP cows. Among them, *MRTFA*, *PDCD1*, and ENSBTAG00000019474 were reported by Martins et al. as DEGs and negatively associated with odds of pregnancy [21]. While these genes are suggested as key players underlying pregnancy and fertility, they are not acting alone. Thus, we implemented a network approach to better understand their involvement in pregnancy success. Through co-expression analysis, we identified 4382 and 3166 unique genes that showed a coordinated expression pattern with the candidate biomarkers in the P and NP networks. Interestingly, only 123 and 116 genes we have identified from the P and NP networks were previously reported as DEGs by Martins et al. [21]. 

Among the candidate biomarkers, *SERPINE3* was more expressed in pregnant cows. Its role in female fertility, however, is unknown. Serpins are a superfamily of protease inhibitors involved in several biological processes [48], including inflammation and tissue remodeling [49]. In line with the same family, the *SERPINE2* gene was highly expressed in the granulosa cells of growing dominant bovine follicles [50]. The *PDCD1* gene has been linked to the growth and differentiation of uterine epithelium [51]. Additionally, this gene codes for an immune-inhibitory receptor important for the maternal immune system during pregnancy [52]. Although we did not find immune-related processes over-represented in our study, Martins et al. [21] reported downregulation of genes associated with immune function in pregnant cows. Other studies in crossbred heifers have reported a cross-talk between immune function and pregnancy outcome [10,53]. The regulatory mechanisms that underlie this interplay, however, remain unclear. 

We identified the *MEF2B* and *MRTFA* transcription factor (TF) coding genes among the predictors of pregnancy outcomes. Both TFs are involved in cell differentiation [54,55]. Additionally, *MEF2* genes code essential regulators of organogenesis [55]. During early pregnancy [56], the MEF2B protein was highly expressed in primary human cytotrophoblasts. Li et al. suggested that MEF2B regulates the extravillous cytotrophoblast invasion and differentiation [56]. *MEF2B* and *MRTFA* were negatively correlated with the *SRF* gene in the network of NP cows. MRTFs co-regulate SRF and activate genes involved in cytoskeletal dynamics and focal adhesion proteins [54,57]. SRF is an important regulator of early development, and its knockout leads to embryonic lethality [58]. We found ENSBTAG00000019474 co-expressed with genes involved in tissue remodeling, vasculogenesis, and embryo development. Interestingly, these processes were over-represented only in the network of NP cows. Likewise, it was more expressed in NP cows. Multiple signaling pathways are required to establish and maintain pregnancy. Considering the biological processes and KEGG pathways we identified, endometrial morphology and remodeling seem to be crucial to proper embryo implantation [59]. 

Although many gene connections were identified in the P network, we found that *SERPINE3*, *FNDC1*, *MRTFA*, *MEF2B*, and ENSBTAG00000019474 were more connected in the NP network. Similar findings were reported by Banerjee et al. [60]. Their study identified a rewiring of major gene regulators in the blood transcriptome network of NP crossbred heifers. These findings suggest that these genes may change regulatory patterns between P and NP cows, consequently leading to deregulated biological pathways [16]. Although our co-expression analysis framework cannot confer information about causality, we found several KEGG pathways that were exclusive to each group. The ribosome pathway was over-represented by co-expressed genes from the P network. Ribosomes are critical for cellular function and metabolism as they control the translation of specific mRNAs [61]. Deficiencies in genes coding to ribosomal proteins and translation initiation factors were related to blastocyst implantation failure [62]. Likewise, based on a proteomics study, Xin et al. identified dysregulation of focal adhesion and ribosome pathways associated with early pregnancy loss in humans [63]. Focal adhesion was over-represented by the shared genes underlying networks from both groups. These molecules play a key role as structural cellular components in integrin-mediated signal transductions and angiogenesis [64]. Previous studies have also highlighted that adhesion molecules are critical during embryo implantation [44,65,66]. 

## 5. Conclusions

We applied a multi-tiered approach to identify predictive candidate biomarkers and fertility-related co-expressed gene networks. Based on that, we identified nine biologically relevant genes expressed in the endometrial epithelium that could discriminate against pregnancy in cows. These genes act in critical pathways for uterine receptivity, including endometrial tissue remodeling, focal adhesion, and embryo development. Furthermore, we identified differences in the network topology of biomarker co-expressed genes between pregnant and non-pregnant cows. In summary, our findings provided new insights into the regulatory network of fertility-related processes. We also demonstrated the potential of combining different analytical tools to prioritize candidate genes and shed light on molecular features involved with pregnancy outcomes. Further investigation, however, is still needed to determine the reliability and sensitivity of these genes in other larger cohorts. Similarly, future experimental studies are needed to elucidate the mechanisms that underlie these biomarkers and their co-expressed pairs in determining pregnancy and fertility.

## Figures and Tables

**Table 1:** Uterine luminal epithelial differentially connected genes between pregnant (P) and non-pregnant (NP) cows.

Ensembl Gene ID	Gene Symbol	Nodes in NP	Nodes in P	DIFFK	z-Score *
ENSBTAG00000001818	*MEF2B*	794	169	0.88983	45.428
ENSBTAG00000003938	*FNDC1*	670	342	0.62088	31.6887
ENSBTAG00000005284	*SERPINE3*	646	401	0.55219	28.1798
ENSBTAG00000019474	*ENSBTAG00000019474*	577	507	0.39619	20.2104
ENSBTAG00000002630	*MRTFA*	373	127	0.38698	19.74
ENSBTAG00000038251	*NAA16*	384	1488	−0.4864	−24.876
ENSBTAG00000020726	*ARHGEF7*	331	1534	−0.5831	−29.818

NP—non-pregnant; P—pregnant; DIFFK—Differential connectivity index. * *p-value* < 0.05.

## Data Availability

All relevant data are within the paper and its Supplementary Information files. All sequencing data is publicly available on Gene Expression Omnibus (GSE171577).

## Supplementary Materials

The following supporting information can be downloaded at: https://www.mdpi.com/article/10.3390/ani12192715/s1, Table S1: List of expressed genes used as background for functional analysis. Table S2: Summary of RNA sequencing and mapping statistics. Table S3: Models generated by BioDiscML. Table S4: Candidate biomarkers and co-expressed pairs identified through the PCIT approach. Table S5: Differentially connected genes identified between P and NP networks. Table S6: KEGG pathway over-representation analysis with significant co-expressed genes of pregnant beef cows. Table S7: KEGG pathway over-representation analysis with significant co-expressed genes of non-pregnant beef cows. Table S8: Over-represented biological processes of individual subnetworks of genes co-expressed with the ENSBTAG00000019474 gene from pregnant cows. Table S9: Over-represented biological processes of individual subnetworks of genes co-expressed with the ENSBTAG00000019474 gene from non-pregnant cows.
